# SIRT1/3 Activation by Resveratrol Attenuates Acute Kidney Injury in a Septic Rat Model

**DOI:** 10.1155/2016/7296092

**Published:** 2016-11-28

**Authors:** Siqi Xu, Youguang Gao, Qin Zhang, Siwei Wei, Zhongqing Chen, Xingui Dai, Zhenhua Zeng, Ke-seng Zhao

**Affiliations:** ^1^Guangdong Key Laboratory of Shock and Microcirculation Research, Department of Pathophysiology, Southern Medical University, Guangzhou 510515, China; ^2^Department of Anesthesiology, The First Affiliated Hospital of Fujian Medical University, Fuzhou, Fujian Province, China; ^3^Department of Critical Care Medicine, Nanfang Hospital, Southern Medical University, Guangzhou 510515, China; ^4^Department of Critical Care Medicine, Institute of Translational Medicine, The First People's Hospital of Chenzhou, Hunan, Chenzhou, China

## Abstract

Sepsis often results in damage to multiple organ systems, possibly due to severe mitochondrial dysfunction. Two members of the sirtuin family, SIRT1 and SIRT3, have been implicated in the reversal of mitochondrial damage. The aim of this study was to determine the role of SIRT1/3 in acute kidney injury (AKI) following sepsis in a septic rat model. After drug pretreatment and cecal ligation and puncture (CLP) model reproduction in the rats, we performed survival time evaluation and kidney tissue extraction and renal tubular epithelial cell (RTEC) isolation. We observed reduced SIRT1/3 activity, elevated acetylated SOD2 (ac-SOD2) levels and oxidative stress, and damaged mitochondria in RTECs following sepsis. Treatment with resveratrol (RSV), a chemical SIRT1 activator, effectively restored SIRT1/3 activity, reduced acetylated SOD2 levels, ameliorated oxidative stress and mitochondrial function of RTECs, and prolonged survival time. However, the beneficial effects of RSV were greatly abrogated by Ex527, a selective inhibitor of SIRT1. These results suggest a therapeutic role for SIRT1 in the reversal of AKI in septic rat, which may rely on SIRT3-mediated deacetylation of SOD2. SIRT1/3 activation could therefore be a promising therapeutic strategy to treat sepsis-associated AKI.

## 1. Introduction

Sepsis is a frequently fatal condition characterized by uncontrolled and adverse host reactions to microbial infection [1], accounting for more than $20 billion (5.2%) of total US hospital costs in 2011 [2]. Sepsis is, by conservative estimates, a leading cause of mortality and critical illness worldwide [3, 4]; however, its pathophysiology remains unclear. The current consensus is that the development of sepsis is characterized by multiple organ dysfunction [1]. The kidney is frequently affected during sepsis, and acute kidney injury (AKI) is a common occurrence during the pathogenesis of sepsis [5]. Tubular cells exhibit reduced oxygen consumption in response to sepsis, indicating severe mitochondrial dysfunction (MD) [6]. Furthermore, our previous studies have shown that severe MD in renal tubular epithelial cells accelerates AKI in a rat model of sepsis [7].

There is increasing evidence that silent mating type information regulation 2 homolog 1 (sirtuin 1, SIRT1) plays an important role in mitochondrial protection. Through deacetylation of histone and nonhistone substrate, SIRT1 is involved in various metabolic and inflammatory diseases [8, 9]. Interestingly, SIRT1 activity is decreased in the liver, spleen, small bowel, and lung tissue in experimental sepsis models, and SIRT1 activation could improve the outcome of sepsis and ameliorate the associated inflammatory response [10, 11]. Besides SIRT1, another sirtuin, SIRT3, has also received considerable attention [12, 13]. Several reports indicate that SIRT3 is exclusively located in the mitochondria and acts as an antioxidative enzyme [12]. In a previous study, our group found that activation of SIRT1/3 improves vascular hyporeactivity in severe hemorrhagic shock by alleviation of mitochondrial damage [14]. However, the role of SIRT1/3 on mitochondrial protection following sepsis is not reported. In this study, we used a septic rat model to determine the effects of SIRT1 and SIRT3 on acute kidney injury (AKI) following sepsis.

## 2. Materials and Methods

### 2.1. Reagents and Antibodies

SOD2 Activity kit was purchased from Dojindo (Kumamoto, Japan). Antibodies against SOD2 and acetylated superoxide dismutase 2 (ac-SOD2) as well as SIRT1 and SIRT3 Deacetylase Fluorometric Assay kits were obtained from Cyclex (Nagano, Japan). Antibody against SIRT1 was purchased from Santa Cruz Biotechnology (Santa Cruz, CA, USA). Antibody against SIRT3 was purchased from ABClonal (Boston, MA, USA). Antibodies against acetylated lysine and glyceraldehyde 3-phosphate dehydrogenase (GAPDH) were purchased from CST (Danvers, MA, USA). Immunoprecipitation kits were obtained from Proteintech (Chicago, IL, USA). Membrane-permeant JC-1 dye and calcein-AM were purchased from Molecular Probes (Eugene, OR, USA). Assay kits for reduced glutathione/oxidized glutathione (GSH/GSSG) and catalase (CAT) were obtained from Beyotime Biotech (Beijing, China). A CellTiter-Glo® Assay kit and terminal deoxynucleotidyl transferase dUTP nick-end labeling (TUNEL) staining kit were purchased from Promega (Madison, WI, USA). Polyvinylidene fluoride (PVDF) membranes were obtained from Millipore (Billerica, MA, USA). 3-(1H-1,2,3-Triazol-4-yl)pyridine (3-TYP), a selective inhibitor of SIRT3, was synthesized and characterized by the School of Pharmaceutical Sciences, Southern Medical University, Guangzhou, China, based on our previous work [15]. All other chemicals were purchased from Sigma-Aldrich (Saint Louis, MO, USA).

### 2.2. CLP Model of Sepsis

The present study was carried out in strict accordance with the recommendations in the Guide for the Care and Use of Laboratory Animals (US National Institutes of Health, Bethesda, MD, USA). The study protocol was approved by the Committee on Ethics in Animal Experiments of Southern Medical University.

In total, 64 specific pathogen-free Sprague-Dawley rats (male or female) weighing 180–220 g were used in this study. The rats were housed in plastic cages with a controlled temperature of 25°C, humidity of 50–55%, and a 12 h light/dark cycle. All the animals had free access to food and distilled water. All rats were anesthetized and maintained with isoflurane (RWD Life Science, Shenzhen, China) and were randomly divided into six groups: the control, vehicle, resveratrol (RSV), SRT1720 (a selective activator of SIRT1), RSV+Ex527, and RSV+3-TYP groups.

1. In the control group, rats were anesthetized and underwent surgery without any other treatment.
2. In the vehicle group, the rats were given vehicle (0.3 mL) and subjected to cecal ligation and puncture (CLP) after 30 min.
3. In the RSV group, the rats were given RSV (0.3 mL; 50 mg/kg) and subjected to CLP after 30 min.
4. In the SRT1720 group, the rats were given SRT1720 (0.3 mL; 0.2 mg/kg) [16] and subjected to CLP after 30 min.
5. In the RSV+Ex527 group, rats were given RSV+Ex527 (0.3 mL, 50 mg/kg, and 5 mg/kg, resp.) and subjected to CLP after 30 min.
6. In the RSV+3-TYP group, the rats were given RSV+3-TYP (0.3 mL, 50 mg/kg, and 5 mg/kg, resp.) [15] and subjected to CLP after 30 min.

Sepsis was introduced by using the CLP technique. Specifically, midline laparotomy was performed using minimal dissection, and the cecum was ligated just below the ileocecal valve by using 4-0 silk ligatures to maintain intestinal continuity. The cecum was perforated at 2 locations 1 cm apart by using an 18-gauge needle and gently compressed until the feces were extruded. The bowel was then returned to the abdomen and the incision closed. Control rats underwent the same surgical procedures, but the cecum was neither ligated nor punctured. At the end of the operation, all rats were subcutaneously resuscitated with vehicle (normal saline, NS; 20 mL/kg). All rats were deprived of food but had free access to water after the operation. All rats received subcutaneous injections of imipenem/cilastatin (14 mg/kg) in 8 mL NS solution (40 mL/kg) at 6 h after CLP.

After the CLP model was created, 36 rats (6 in each group) were killed for tissue extraction, observation of mitochondrial morphology, and renal tubular epithelial cell (RTEC) isolation; the remaining animals (8 in each group) were used for survival analyses.

### 2.3. RTEC Isolation

A portion of extracted renal tissue from each group was used for isolation of RTECs by our previously described methods [7]. Briefly, the cortex was cut into fragments, and cells were dissociated by incubation with 1 mg/mL type-I collagenase for 30 min at 37°C. Red blood cells were removed by lysis. RTECs were separated by Percoll gradient density centrifugation. The purity of the RTECs was determined by immunostaining with cytokeratin-18 and Hoechst dye.

### 2.4. Western Blotting

Both kidney tissue and isolated RTECs were centrifuged at 14000 rpm for 10 minutes after homogenization in Radio-Immunoprecipitation Assay (RIPA) lysis buffer, and the clear supernatants were collected. Total protein concentrations in the supernatants were determined by the bicinchoninic acid (BCA) method. Then, the protein was boiled at 98°C for 5 to 10 minutes and stored at −80°C for later analysis. Equal amounts of protein samples were electrophoresed through a 7.5% SDS-polyacrylamide gel and then transferred onto polyvinylidene difluoride (PVDF) membrane using wet transfer at 100 V for 90 minutes at 4°C. Nonspecific binding sites were blocked by 1% BSA in 0.05% Tween-20 Tris-buffered saline (TBST) and then incubated overnight at 4°C with primary antibodies. After incubation with primary antibodies (against SIRT1, SIRT3, SOD2, cytochrome C, and GAPDH) and secondary antibodies, protein bands were detected using chemiluminescence detection reagents. GAPDH was used as an internal reference. Ac-SOD2 levels on immunoprecipitated SOD2 protein were measured. Band intensity was quantified by scanning densitometry. Each measurement was made at least 3 times.

### 2.5. SIRT1/3 Activity

Activity of SIRT1 deacetylase was detected using SIRT1 (Cyclex, Cat#CY-1151V2) and SIRT3 Deacetylase Fluorometric Assay kits (Cyclex, Cat#CY-1153V2) as described previously [13]. Briefly, renal tissue samples (50 mg) or RTECs were homogenized in 500 *μ*L immunoprecipitation buffer. After immunoprecipitation of SIRT1/3, final reaction mixtures (50 *μ*L) contained 50 mM Tris-HCl (pH 8.8), 4 mM MgCl_2_, 0.5 mM dithiothreitol, 0.25 mA/mL lysyl endopeptidase, 1 *μ*M trichostatin A, 200 *μ*M NAD^+^, and 5 *μ*L extraction buffer. Fluorescence intensity at 350 nm/450 nm was measured using an Automatic Microplate Reader (Molecular Devices, Sunnyvale, CA, USA). Activity was presented as a relative value compared with that of the control group.

### 2.6. SOD2 Activity

SOD2 activity was measured with a commercially available kit using water-soluble tetrazolium salt- (WST-) 1 as a substrate [14]. Briefly, total SOD activity of each immunoprecipitated protein (normalized to that of the control group) was measured by inhibition of the rate of WST-1 reduction. Potassium cyanide was added to the lysate during the assay to inhibit both SOD1 and SOD3. Absorbance was read at 450 nm using a Microplate Reader (SpectraMax M5). Relative SOD2 activity (compared with that of the control group) is shown.

### 2.7. GSH Content, GSH/GSSG Ratio, and CAT Activity

GSH, the GSSG/GSH ratio, and CAT activity in isolated RTECs were evaluated using kits according to manufacturer instructions and standard methods. Briefly, 200 *μ*L of homogenized cell samples was mixed with the reagent provided in the kit and was processed in boiled water and then ice as stated in the instruction. Absorbance at 520 nm was obtained for GSH and GSSG by a Microplate Reader, and the concentrations of these two enzymes were calculated. For CAT, absorbance at 520 nm was obtained on a UV spectrophotometer. The relative values of CAT compared to the control group were then calculated.

### 2.8. Mitochondrial Morphology

All rats (6 in each group) were killed and the renal tissue was used for morphological observation and protein extraction. Morphological changes in the mitochondria of cells from renal tissue were observed using transmission electron microscopy. Renal tissues were fixed with 2.5% glutaraldehyde and stained with cacodylate-buffered osmium tetroxide. Sections were prepared and examined under an electron microscope (H-7500; Hitachi, Tokyo, Japan) [17].

### 2.9. Evaluation of Mitochondrial Function

Isolated cells were used for the detection of mitochondrial function (mitochondrial membrane potential (ΔΨm), cellular level of adenosine triphosphate (ATP), and mitochondrial permeability transition pore (mPTP)), as described previously. Mitochondrial function-related indices such as the ΔΨm (using membrane-permeant JC-1 dye) and mPTP were analyzed using a Flow Cytometer (FACSVerse; Becton Dickinson, San Jose, CA, USA). The aggregates-to-monomers ratio of JC-1 represented the ΔΨm. Fluorescence intensity reflected the status of opening of the mPTP. ATP content was measured by an assay that measures ATP through the energy-dependent luciferase/luciferin reaction and provides information on cell viability. The test was performed according to manufacturer instructions. After counting cells, 100 *μ*L CellTiter-Glo was added to a cell suspension (100 *μ*L) containing 10,000 isolated cells in each well of a standard opaque-walled 96-well plate. Plates were incubated at room temperature for 10 min, and luminescence was recorded in a SpectraMax M5 Microplate Reader (Molecular Devices, Sunnyvale, CA) [7].

### 2.10. TUNEL Apoptosis Assay

For detection of apoptotic cells, TUNEL staining was carried out using a Promega apoptosis detection kit. Immunofluorescence for TUNEL staining was performed with fluorescein isothiocyanate (FITC). The glass was mounted with cover slips containing 4′,6-diamidino-2-phenylindole (DAPI) and imaged under a Confocal Microscope (LSM 780; Carl Zeiss, Oberkochen, Germany). Cells stained green fluorescence and DAPI stained blue fluorescence were detected as TUNEL-positive apoptotic cells. Both TUNEL-positive and DAPI-positive cells were counted in 10 random high-power fields (HPF; 300-cells each). Data are expressed as the number of apoptotic cells/HPF (400x magnification).

### 2.11. Animal Survival

Some of the animals in each group (32 rats in total, 8 in each group) were assigned to a subgroup for survival analyses. To minimize suffering, pentobarbital sodium (30 mg/kg, i.p.) was given intermittently to conscious animals. Animals had access to food and water* ad libitum*. Apnea for >1 min was considered to indicate death. At the end of CLP, catheters were removed and skin wounds were sutured. Survival time and the prevalence of survival at 48 h were recorded. Rats that survived for >7 d were killed by cervical dislocation.

### 2.12. Statistical Analyses

Median survival was analyzed using Kaplan–Meier plots and compared using the log-rank test. Other data were presented as the mean ± standard deviation and were analyzed using SPSS 20.0 (IBM, Armonk, NY, USA). Levene's test was used to ascertain if groups had equal variance. Moreover, one-way analysis of variance (ANOVA) followed by Tukey's test was applied. If equal variances were not assumed (based on Levene's test; *p* < 0.1), Dunnett's T3* post hoc* comparisons were used for robust tests of equality of mean values. Level of significance was set at *p* < 0.05.

## 3. Results

### 3.1. SIRT1 Activity Was Decreased in Renal Tissue following CLP

**Figure 1:**
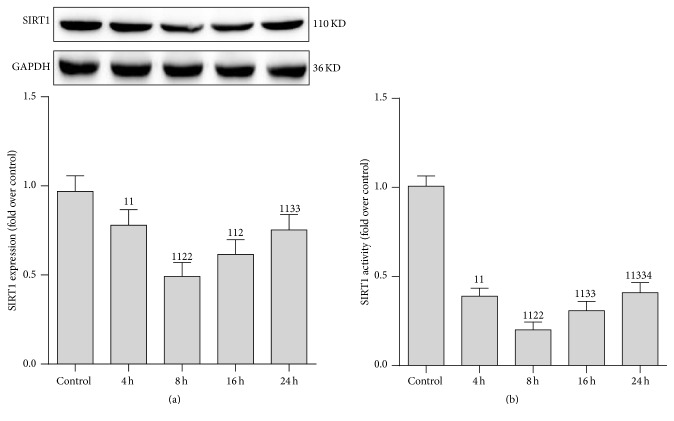
Expression and activity of SIRT1 protein in renal tissue after cecal ligation and puncture (CLP). (a) Representative western blot of SIRT1 proteins (upper panel) and densitometric analyses (lower panel); (b) SIRT1 activity was determined using a SIRT1 Assay kit and normalized to that of the control group. The time points were set at 4, 8, 16, and 24 h after CLP treatment. Values shown are the mean ± SEM. ^11^ *p* < 0.01 versus the control group; ^2^ *p* < 0.05 and ^22^ *p* < 0.01 versus the 4 h group; ^33^ *p* < 0.01 versus the 8 h group; ^4^ *p* < 0.05 versus the 16 h group. *N* = 6. SIRT1, sirtuin; GAPDH, glyceraldehyde 3-phosphate dehydrogenase.

To determine whether SIRT1 is involved in the pathogenesis of sepsis-induced AKI, we studied both the activity and protein expression of SIRT1 in renal tissue (Figure 1). The rats were killed 4, 8, 16, and 24 h after CLP treatment and AKI was confirmed as previously described [18]. As expected, SIRT1 protein expression and activity, particularly SIRT1 activity, progressively reduced 8 h after CLP. After that, both protein expression and activity were partially restored. Therefore, 8 h after CLP treatment was selected as the time point in the follow-up study.

### 3.2. SIRT1 Activity Was Decreased in RTECs following CLP

We then explored which kind of renal cell contributed to the decreased SIRT1 activity. Both the RTECs and glomerular cells were isolated immediately after the rats were executed. To further test the role of SIRT1 in AKI induced by CLP, the SIRT1 chemical activator RSV and SRT1720 and inhibitor Ex527 were used. SIRT1 protein expression and activity were considerably reduced in RTECs compared to that in glomerular cells (see Supplementary Figure 1 in Supplementary Material available online at http://dx.doi.org/10.1155/2016/7296092).

**Figure 2:**
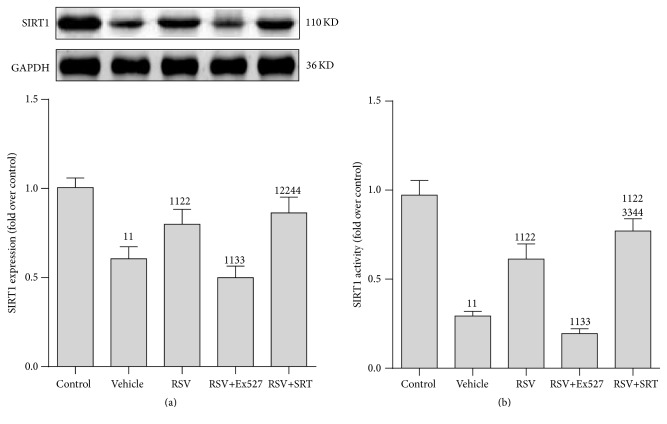
Expression and activity of SIRT1 protein in renal tubular epithelial cells after CLP. (a) Representative western blot of SIRT1 proteins (upper panel) and densitometric analyses (lower panel); (b) SIRT1 activity was determined using a SIRT1 Assay kit and normalized to that of the control group. Values shown are the mean ± SEM. ^11^ *p* < 0.01 versus the control group; ^22^ *p* < 0.01 versus the vehicle group; ^33^ *p* < 0.01 versus the RSV group; ^44^ *p* < 0.01 versus the RSV+SRT group. *n* = 6. RSV, resveratrol; SIRT1, sirtuin; GAPDH, glyceraldehyde 3-phosphate dehydrogenase; RSV, resveratrol; SRT, SRT1720. ^1^presents the mean grey density value of protein bands of control group.

Furthermore, RSV greatly restored SIRT1 activity and slightly elevated the SIRT1 protein expression. Interestingly, SRT1720 addition increased the SIRT1 activity slightly more than RSV treatment alone. In contrast, the beneficial effect of RSV was blocked after Ex527 was added (Figure 2).

### 3.3. SIRT3 Activity Was Reduced in RTECs following CLP

**Figure 3:**
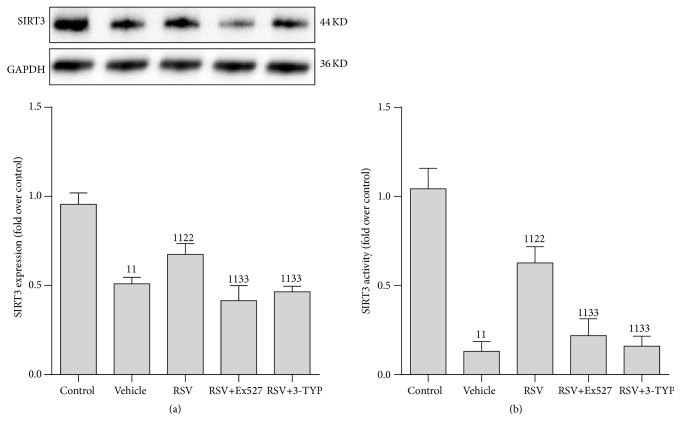
Expression and activity of SIRT3 protein in renal tubular epithelial cells after CLP. (a) Representative western blot of SIRT3 proteins (upper panel) and densitometric analyses (lower panel); (b) SIRT3 activity was determined using a SIRT3 Assay kit and normalized to that of the control group. Values shown are the mean ± SEM. ^11^ *p* < 0.01 versus the control group; ^22^ *p* < 0.01 versus the vehicle group; ^33^ *p* < 0.01 versus the RSV group. *n* = 6. SIRT3, sirtuin 3; GAPDH, glyceraldehyde 3-phosphate dehydrogenase; RSV, resveratrol; 3-TYP, 3-(1H-1,2,3-triazol-4-yl)pyridine.

Based on the importance of the SIRT1/3 axis in the pathogenesis of severe shock [14], we hypothesized that SIRT3 may play a role in sepsis. SIRT3 expression and activity in RTECs were tested. Consistent with our observations regarding SIRT1, the SIRT3 expression and activity were reduced following CLP and were restored by RSV treatment; however, the beneficial effect of RSV on SIRT3 was either abrogated by Ex527 (a selective inhibitor of SIRT1) or 3-TYP (a selective inhibitor of SIRT3) (Figure 3).

### 3.4. SIRT1 Activation Restored SOD2 Activity in RTECs following CLP

**Figure 4:**
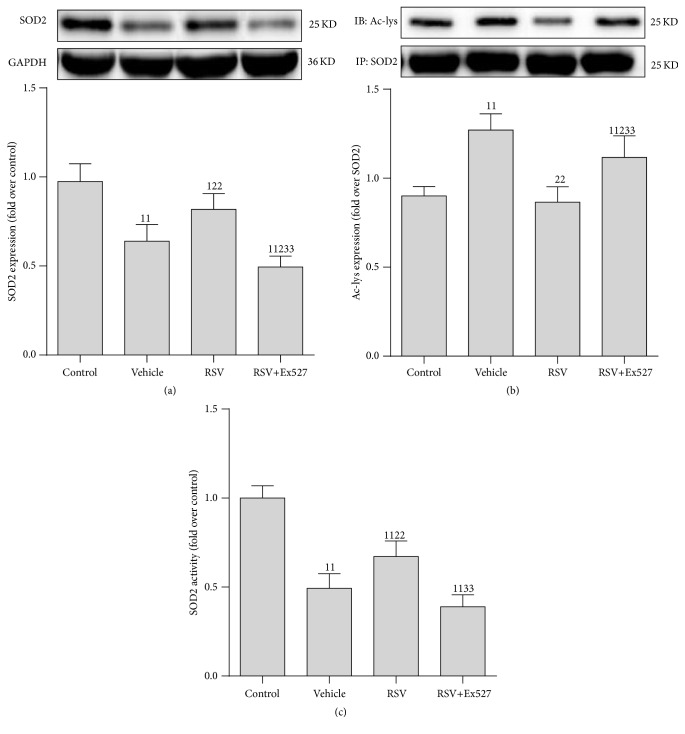
Expression of SOD2 protein, acetylation level, and activity in renal tubular epithelial cells after CLP. (a) Representative western blot of SOD2 proteins (upper panel) and densitometric analyses (lower panel); (b) level of acetylated SOD2 was determined using an acetylated SOD2 (Ac-SOD2) antibody normalized to purified SOD2 protein by immunoprecipitation (IP SOD2); (c) SOD2 activity was determined using a SOD2 Assay kit and normalized to that of the control group. Results are expressed as fold change over control. Values shown are the mean ± SEM. ^1^ *p* < 0.05 and ^11^ *p* < 0.01 versus the control group; ^2^ *p* < 0.05 and ^22^ *p* < 0.01 versus the vehicle group; ^33^ *p* < 0.01 versus the RSV group. *n* = 6. RSV, resveratrol; Ac-lys, acetylated lysine; GAPDH, glyceraldehyde 3-phosphate dehydrogenase; SOD2, superoxide dismutase 2.

Since several reports claim that SOD2 is one of the deacetylated targets of SIRT1/3, we determined protein expression, acetylation level, and activity of SOD2 in RTECs following CLP. As expected, RSV treatment slightly restored the SOD2 protein content and remarkably decreased the acetylated SOD2 level (Ac-lys), leading to elevated SOD2 activity (Figure 4).

### 3.5. SIRT1 Activation Ameliorated Oxidative Stress in RTECs following CLP

**Figure 5:**
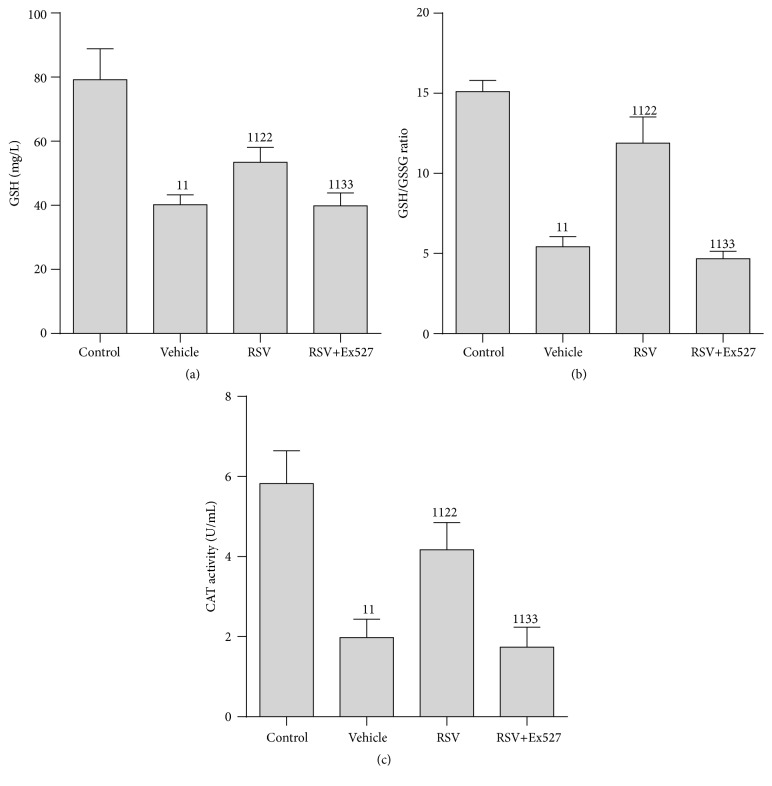
Indices of oxidative stress in the renal tubular epithelial cell after CLP. (a) GSH content; (b) GSH/GSSG ratio; (c) CAT activity. Results are expressed as fold change over control. Values shown are the mean ± SEM. ^11^ *p* < 0.01 versus the control group; ^22^ *p* < 0.01 versus the vehicle group; ^33^ *p* < 0.01 versus the RSV group. *n* = 6. RSV, resveratrol; GSH, reduced form of glutathione; GSSG, oxidized form of glutathione; CAT, catalase.

Since SOD2 activity was significantly restored by SIRT1 activation and SOD2 is a key antioxidative enzyme, we tested GSH content, GSH/GSSG ratio, and CAT activity in RTECs. The GSH content, GSH/GSSG ratio, and CAT activity were considerably decreased after CLP in the vehicle group and were partially restored by SIRT1 activation in the RSV treatment group; however, after Ex527 was added, the beneficial effect of RSV was greatly blocked and was almost equivalent to that in the vehicle group (Figure 5).

### 3.6. SIRT1 Activation Attenuated MD in RTECs following CLP

**Figure 6:**
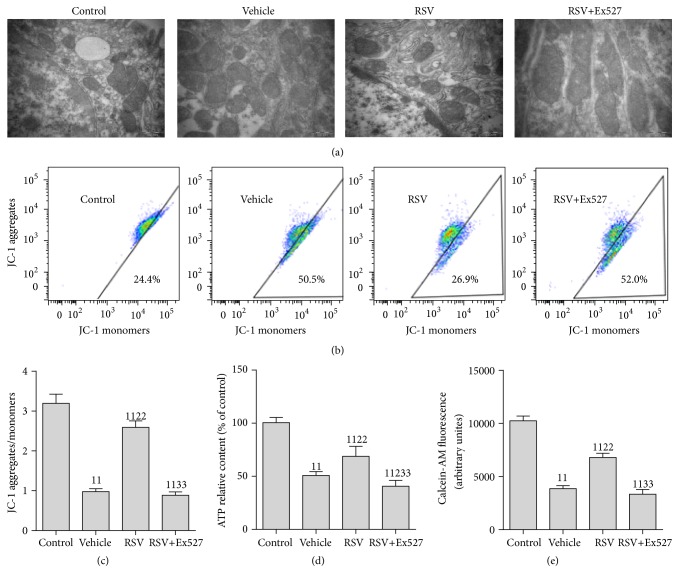
Mitochondrial morphology and function of renal tubular epithelial cells after CLP. (a) Representative transmission electron microscope images of the mitochondria. Healthy mitochondria (control group) have intact mitochondrial membranes and cristae. Mitochondria are swollen, with poorly defined cristae and more mitochondrial vacuolization in the vehicle group, whereas these changes are prevented partially in RSV group. (b) Representative flow cytometry scatter plots of the JC-1 probe. Cells were stained with the JC-1 probe; regions of reduced polarization are indicated by the JC-1 monomers. Regions of high polarization of mitochondrial membranes are indicated by the formation of J-aggregates. (c) ΔΨm was monitored using JC-1 by flow cytometry and mitochondrial depolarization was quantified as JC-1 aggregates/monomers. (d) ATP level was determined using a System Bioluminescence Detection kit. (e) Flow cytometric quantification of calcein-AM fluorescence. Intensity of calcein-AM fluorescence reflects the extent of opening of the mPTP. Values are presented as the mean ± SEM. ^11^ *p* < 0.01 versus the control group; ^2^ *p* < 0.05 and ^22^ *p* < 0.01 versus the vehicle group; ^33^ *p* < 0.01 versus the RSV group. *n* = 6. RSV, resveratrol; ATP, adenosine triphosphate; ΔΨm, mitochondrial membrane potential.

Due to the positive effect of SIRT1 activation on SOD2, an antioxidative stress enzyme located in the mitochondria, we also tested the mitochondrial morphology and function in RTECs. An elliptical shape with well-developed cristae and electron-dense matrices was noted in the control group. Severe MD was found after CLP treatment (vehicle group), evidencing by irregularly shaped, swollen, and disrupted mitochondria with poorly defined cristae and electron-lucent matrices. Surprisingly, these changes were restored by treatment with RSV. Moreover, RSV treatment restored the mitochondrial transmembrane potential (JC-1 aggregates/monomer), enhanced ATP content, and inhibited the opening of mPTP. However, these beneficial effects of RSV were considerably blocked by Ex527 addition (Figure 6).

### 3.7. SIRT1 Activation Inhibited Apoptosis in RTECs

**Figure 7:**
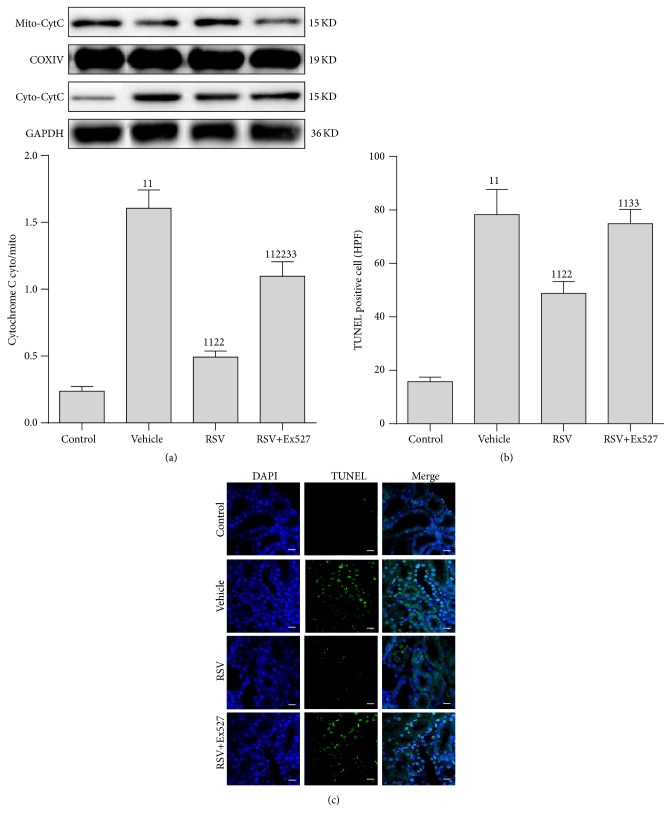
Cytochrome C expression and TUNEL staining of apoptotic cells in the kidneys of rats after CLP. (a) Representative western blot of cytochrome C protein in mitochondria (mito-CytC) and cytoplasm (cyto-CytC). (b) Densitometric analyses of cytochrome C protein in mitochondria (mito-CytC) and cytoplasm (cyto-CytC). (c) TUNEL staining. Original magnification ×400. *n* = 6. Values shown are mean ± SEM. ^11^ *p* < 0.01 versus the control group; ^22^ *p* < 0.01 versus the vehicle group; ^33^ *p* < 0.01 versus the RSV group. *n* = 6. DAPI, 4′,6-diamidino-2-phenylindole; TUNEL, terminal deoxynucleotidyl transferase dUTP nick-end labeling; RSV, resveratrol.

We subsequently tested the effect of SIRT1 activation on apoptosis in RTECs; a mitochondria-related apoptosis index (cytochrome C) and a general apoptosis index (TUNEL) were selected. The mitochondrial cytochrome C (mito-cyt C) was decreased and the cytoplasmic cytochrome C (cyto-cyt C) was increased after CLP treatment. Moreover, the number of TUNEL-positive cells was significantly increased. As expected, RSV administration reduced the cyto-cyt C content and decreased the number of TUNEL-positive cells. However, the beneficial effects of RSV were reversed considerably by Ex527 addition (Figure 7).

### 3.8. SIRT1 Activation Protected Renal Function and Prolonged Survival Time in Septic Rat

Finally, we tested the effect of SIRT1 activation on renal function and survival time. Both the creatinine and urea nitrogen were elevated in septic rat, accompanied by shortened survival time. SIRT1 activation by RSV considerably reduced the level of creatinine and blood urea nitrogen. Importantly, RSV administration greatly prolonged the survival of sepsis animal. However, the beneficial effect of RSV on renal function and survival time were partially abrogated by Ex527 (Supplementary Figure 2).

## 4. Discussion

Our results showed that SIRT1/3 activity was reduced in the RTECs of septic rat and was accompanied by higher acetylated SOD2 levels, swollen mitochondria, and increased cell apoptosis. Moreover, SIRT1 activation by RSV could partially restore SIRT3 activity and attenuate MD, leading to improved mitochondrial function and reduced apoptosis. Furthermore, all the beneficial effects of SIRT1 activation were considerably reduced by a selective inhibitor. To the best of our knowledge, this is a first report describing a role for SIRT1/3 in the pathogenesis of sepsis-associated AKI.

SIRTs, first discovered in yeast as NAD^+^-dependent epigenetic and metabolic regulators, have comparable activities in human physiology and disease [9]. There is increasing evidence that SIRT1 mediates protein posttranslational modification related to aging and ischemic disease [19–21]. In our previous studies, we showed that SIRT1 activation could ameliorate MD in hepatocytes [22], vascular smooth muscle cells [23], small intestine epithelial cells, and RTECs [17] in a severe hemorrhagic shock rat model, consistent with reports of the beneficial effects of SIRT1 in ischemic conditions. However, the role of SIRT1 in sepsis is rarely studied and its exact mechanism is yet to be determined. Recently, a study showed that SIRT1 knockdown mice exhibited aggravated inflammatory signaling such as NF-kappa B in lung tissue of CLP mice [10]. Furthermore, pharmacological SIRT1 activation decreased mortality in experimental sepsis [24]. Importantly, RSV was found to effectively inhibit inflammatory responses by acting as a SIRT1 activator [25]. Some studies have shown that HMGB1 is a main downstream deacetylated target of SIRT1 [26] and SIRT1 activation inhibited HMGB1 secretion [27]. In contrast, some reports have claimed that SIRT1 inhibition, but not activation, could be a novel way to treat sepsis in its hypoinflammatory phase [28]. Liu et al. used TLR4-stimulated THP1 human promonocytes to mimic the adaptation stages of sepsis. They found that nuclear SIRT1 guides RELB to differentially induce SIRT3 expression and increase mitochondrial biogenesis. Based on their results, they claimed that SIRT1 inhibition repressed bioenergetics during sepsis adaptation and finally improved survival time [29]. This seeming discrepancy may suggest that the treatment of sepsis should differ based on the phase of sepsis. In this study, we demonstrated that SIRT1 protein levels and activity in kidney tissue progressively declined in the early phase (−8 h) of sepsis. Even though SIRT1 protein levels partially increased after 8 h, the activity was barely restored at 24 h. The compensatory response of the organism may account for this phenomenon. This result also suggests the importance of timing for therapy. In this study, we used a potential SIRT1 activator, resveratrol, for treatment of sepsis. We found that SIRT1 activation effectively protected mitochondrial function in RTECs and ameliorated renal function (Supplementary Figure 2). However, when a special inhibitor of SIRT1, Ex527, was added, the beneficial effect of RSV was greatly abrogated. These results indicate that SIRT1 is a promising target for pharmacological intervention in septic AKI.

In terms of mitochondrial function, SIRT3 is another known deacetylating enzyme. A growing body of evidence has confirmed that SIRT3 defends against oxidative stress in multiple diseases including ischemia and neurodegenerative disease. Genetic inference or genetic knockout of SIRT3 accelerates diet-induced obesity, type 2 diabetes, and nonalcoholic fatty liver disease [8]. SIRT3 activation can deacetylate its downstream target SOD2 (also termed manganese superoxide dismutase, Mn-SOD), leading to a reduction in oxygen free radicals in the mitochondria and other subcellular organelles [12, 13]. Interestingly, the relationship between the functions of nuclear SIRT1 and mitochondrial SIRT3 is coordinated during the hypoinflammatory phase of sepsis in THP1 human promonocytes [29]. Liu et al. found that SIRT1, RELB, and SIRT3 act in sequence to modify mitochondrial bioenergetics. They proved that the suppression of SIRT1/3 protein expression could improve the survival of septic mice [29]. However, the relative role of each protein in influencing sepsis outcomes and how their programming is coordinated must still be determined. In this study, we did not directly test the effects of SIRT3; instead, we activated or deactivated SIRT1 using chemical agents and found that SIRT3 activity is considerably reduced in the RTECs of septic rat. Moreover, SIRT1 activation by RSV partially restored SIRT3 activity, indicating that SIRT1 might be an upstream regulator of SIRT3 or might provide cooperative protection, decreasing acetylated SOD2 and restoring SIRT3 activity. The protein content of the antioxidative enzyme GSH and activity of CAT were reduced. However, the activation effect of SIRT1 on SIRT3 disappeared when a selective inhibitor targeted at SIRT3, 3-TYP, was added.

Some reports have confirmed that the dysfunction of the renal peritubular microenvironment leads to sepsis-induced AKI [30]. The swelling of RTECs owing to LPS stimulation was found to cause proximal tubule obstruction and reduce tubular flow rate [31]. These reports indicate the vital role of RTECs in kidney function. Further, in our previous report, we demonstrated that MD existed in RTECs in the septic rat [7]. In agreement with the aforementioned studies, we confirmed that RTECs are among the more susceptible cells in kidney tissue. Besides RTECs, we also observed SIRT1 activity in glomerular epithelial cells (Supplementary Figure 1). These results collectively suggest that SIRT1 plays a considerable role in the pathogenesis of septic AKI.

Our study has some potential limitations. Firstly, sepsis is a dynamic disease. It changes from the hyperinflammatory to hypoinflammatory phase. In this study, we only assessed the early (hyperinflammatory) phase of sepsis; further studies regarding therapeutic options during the hypoinflammatory phase are needed. However, this study provides a prevention strategy for sepsis AKI treatment, which partially elucidates the SIRT1-SIRT3 axis in the pathogenesis of sepsis. Secondly, we applied a chemical activator and a selective inhibitor against SIRT1; more intensive methods such as genetic inference or genetic knockout may provide stronger evidence. Thirdly, we only explored the effects of SIRT1 in RTECs; reduction of SIRT1 activity also occurs in other types of kidney cells and will certainly be a worthwhile direction for future studies.

## 5. Conclusion

This study outlines an important role for SIRT1 in the reversal of AKI following sepsis. In addition, our findings suggest that RSV treatment exerts a profound protective effect against renal injury caused by sepsis. This protection appears to be largely due to the ability to activate SIRT1 and SIRT3, augment SOD2-mediated antioxidative ability, and mitochondrial protection.

## Supplementary Material

Supplemental figure 1. Expression and activity of SIRT1 protein in glomerular epithelial cells after CLP. (a). Representative western blot of SIRT1 proteins (upper panel) and densitometric analyses (lower panel); (b) SIRT1 activity was determined using a SIRT1 Assay kit and normalized to that of the control group. Values are presented as the mean ± SEM. 11p < 0.01 vs. the control group; 22p < 0.01 vs. the vehicle group; 33p < 0.01 vs. the RSV group; 44p < 0.01 vs. the RSV+Ex527 group. n = 6. SIRT1, sirtuin; GAPDH, glyceraldehyde 3-phosphate dehydrogenase; RSV, resveratrol; SRT, SRT1720.

## Abbreviations

AKI: Acute kidney injury
RTEC: Renal tubular epithelial cell
CLP: Cecal ligation and puncture
RSV: Resveratrol
ac-SOD2: Acetylated superoxide dismutase 2
SOD: Superoxide dismutase
SIRT: Sirtuin
MD: Mitochondrial dysfunction
GSH: Reduced form of glutathione
GSSG: Oxidized form of glutathione
CAT: Catalase
TUNEL: Terminal deoxynucleotidyl transferase dUTP nick-end labeling
PVDF: Polyvinylidene fluoride
WST: Water-soluble tetrazolium salt
GAPDH: Glyceraldehyde 3-phosphate dehydrogenase
ΔΨm: Mitochondrial membrane potential
ATP: Adenosine triphosphate
mPTP: Mitochondrial permeability transition pore
HPF: High-power field.
