# Supplementary material for: SIRT1/3 Activation by Resveratrol Attenuates Acute Kidney Injury in a Septic Rat Model
Source: Oxid Med Cell Longev. 2016 Nov 28;2016:7296092. doi: 10.1155/2016/7296092 (PMC5149703; doi:10.1155/2016/7296092)
Supplement: Supplementary file 1 — Supplemental figure 1. Expression and activity of SIRT1 protein in glomerular epithelial cells after CLP. (a). Representative western blot of SIRT1 proteins (upper panel) and densitometric analyses (lower panel); (b) SIRT1 activity was determined using a SIRT1 Assay kit and normalized to that of the control group. Values are presented as the mean ± SEM. 11p < 0.01 vs. the control group; 22p < 0.01 vs. the vehicle group; 33p < 0.01 vs. the RSV group; 44p < 0.01 vs. the RSV+Ex527 group. n = 6. SIRT1, sirtuin; GAPDH, glyceraldehyde 3-phosphate dehydrogenase; RSV, resveratrol; SRT, SRT1720. [file 7296092.f1.pptx]

## Slide 1
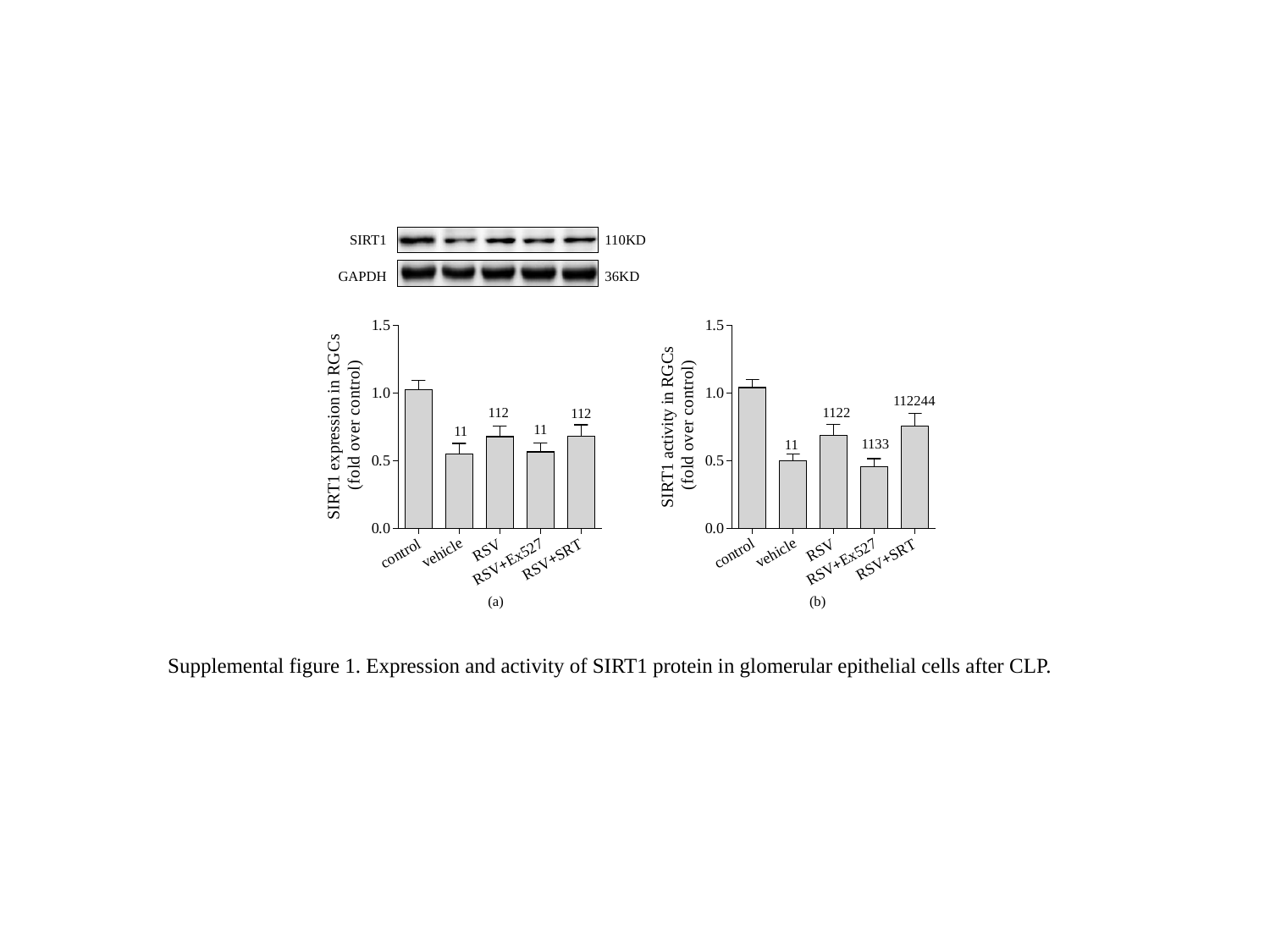

SIRT1
110KD
36KD
GAPDH
112244
112
1122
112
11
11
1133
11
(a)
(b)
Supplemental figure 1. Expression and activity of SIRT1 protein in glomerular epithelial cells after CLP.

## Slide 2
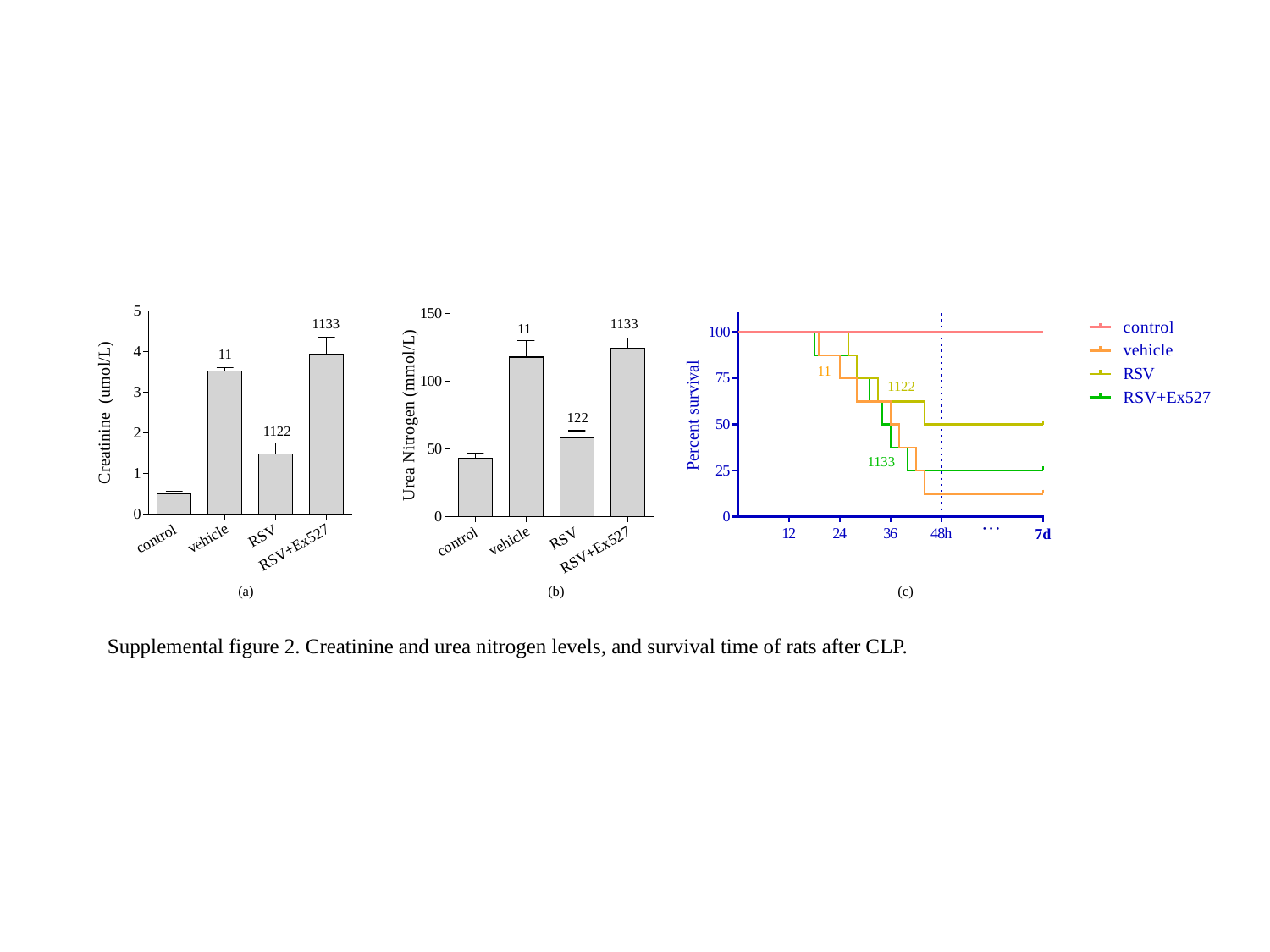

1133
1133
11
11
11
1122
122
1122
1133
···
(a)
(b)
(c)
Supplemental figure 2. Creatinine and urea nitrogen levels, and survival time of rats after CLP.
